# Addendum: Thousands of human non-AUG extended proteoforms lack evidence of evolutionary selection among mammals

**DOI:** 10.1038/s41467-023-44405-6

**Published:** 2024-01-03

**Authors:** Alla D. Fedorova, Stephen J. Kiniry, Dmitry E. Andreev, Jonathan M. Mudge, Pavel V. Baranov

**Affiliations:** 1 School of Biochemistry and Cell Biology, University College Cork, Cork, Ireland; 2 SFI Centre for Research Training in Genomics Data Science, University College Cork, Cork, Ireland; 3 Shemyakin-Ovchinnikov Institute of Bioorganic Chemistry, RAS, Moscow, Russia; 4 Belozersky Institute of Physico-Chemical Biology, Lomonosov Moscow State University, Moscow, Russia; 5 European Molecular Biology Laboratory, European Bioinformatics Institute, Wellcome Genome Campus, Hinxton, Cambridge UK

**Keywords:** Sequence annotation, Genome informatics, Proteome, Molecular evolution, Translation

Addendum to: *Nature Communications* 10.1038/s41467-022-35595-6, published online 23 December 2022

We would like to make readers aware that after the publication of the original article, we found that the parameters for PhyloCSF score calculation were obtained for an alignment other than 120 mammals. This made our analysis less sensitive than intended. Recalculation of the PhyloCSF score with the intended parameters produced a larger dataset of 177 genes that contains 58 of 60 genes in the original dataset are provided in Supplementary Data 1 here. This difference does not alter the main conclusion of the article regarding the substantial prevalence of ribo-seq-supported non-AUG-initiated N-terminal extensions over those supported by phylogenetic analysis. However, it indicates that the number of N-terminally extended proteoforms satisfying our criteria for protein-coding evolution is three times greater than originally reported. The corrected parameters for 120 mammals are now available on the PhyloCSF github page (https://github.com/mlin/PhyloCSF/tree/master/PhyloCSF_Parameters).

### Supplementary information

Supplementary Data 1

