# Supplementary material for: Thousands of human non-AUG extended proteoforms lack evidence of evolutionary selection among mammals
Source: Nat Commun. 2022 Dec 23;13:7910. doi: 10.1038/s41467-022-35595-6 (PMC9789052; doi:10.1038/s41467-022-35595-6)
Supplement: Supplementary file 1 — Supplementary Information [file 41467_2022_35595_MOESM1_ESM.pdf]

Thousands of human non-AUG extended proteoforms lack evidence of evolutionary selection among mammals

Supplementary Figures **2**

Supplementary Methods **12**

Detection of translated regions in Trips-Viz 12

Supplementary Notes **14**

In-frame and out-of-frame AUGs in theoretical extensions of PhyloSET and RiboSET 14

Exclusive non-AUG initiation 15

Supplementary References **16**

Supplementary Figures

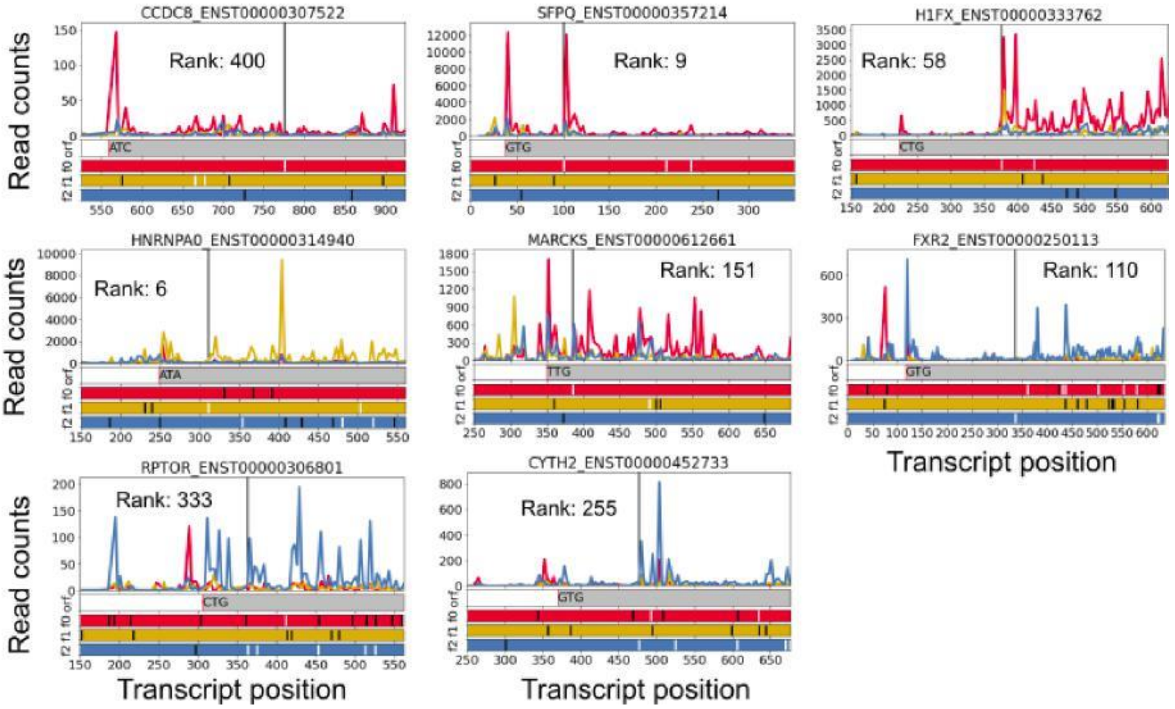

Supplementary Fig.1. Subcodon Ribo-seq profiles of 8 genes with non-AUG extended proteoforms found in both RiboSET and PhyloSET (both CDS and extension are translated). Densities of ribosome footprints differentially coloured based on the supported reading frame. The colours are matched to the reading frames in ORF plot at the bottom where AUG codons are depicted as white and stop codons as black dashes. Black vertical lines indicate the starts of the annotated CDS. Grey bar indicates the predicted extension. The genomic intervals including primary extension and the first at least 250 nucleotides of CDS are shown. Source data is provided as a Source Data file.

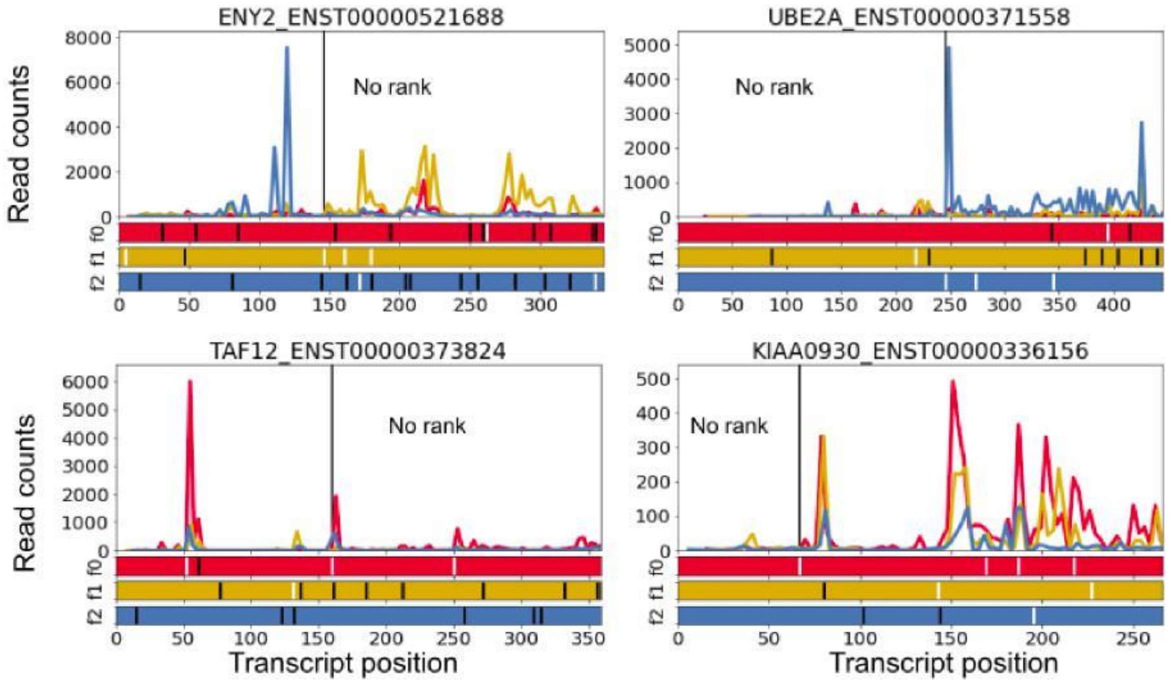

Supplementary Fig.2. Subcodon Ribo-seq profiles of genes in PhyloSET which have no Trips-viz rank with high CDS coverage. Densities of ribosome footprints differentially coloured based on the supported reading frame. The colours are matched to the reading frames in ORF plot at the bottom where AUG codons are depicted as white and stop codons as black dashes. Black vertical lines indicate the starts of the annotated CDS. The genomic intervals including primary extension and the first at least 250 nucleotides of CDS are shown. Source data is provided as a Source Data file.

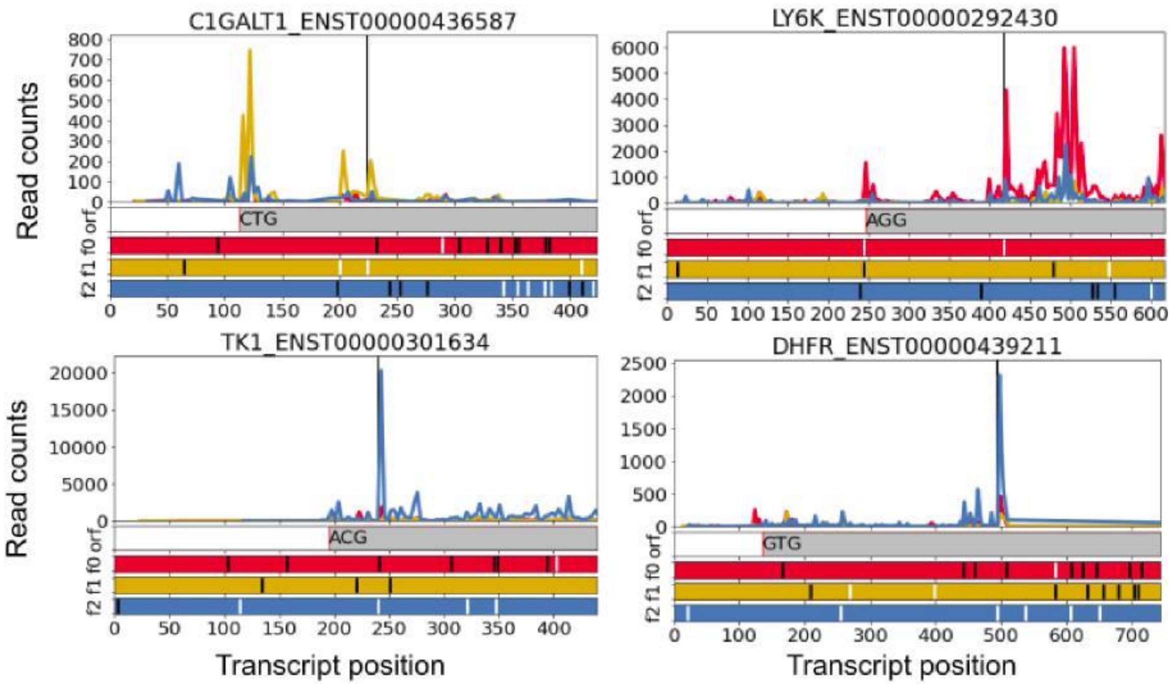

Supplementary Fig.3. Subcodon Ribo-seq profiles of 4 genes with in-frame AUGs found within theoretical extension sequences. Densities of ribosome footprints differentially coloured based on the supported reading frame. The colours are matched to the reading frames in ORF plot at the bottom where AUG codons are depicted as white and stop codons as black dashes. Black vertical lines indicate the starts of the annotated CDS. Yellow rectangle highlights the predicted extension. The genomic intervals including primary extension and the first at least 150 nucleotides of CDS are shown. Source data is provided as a Source Data file.

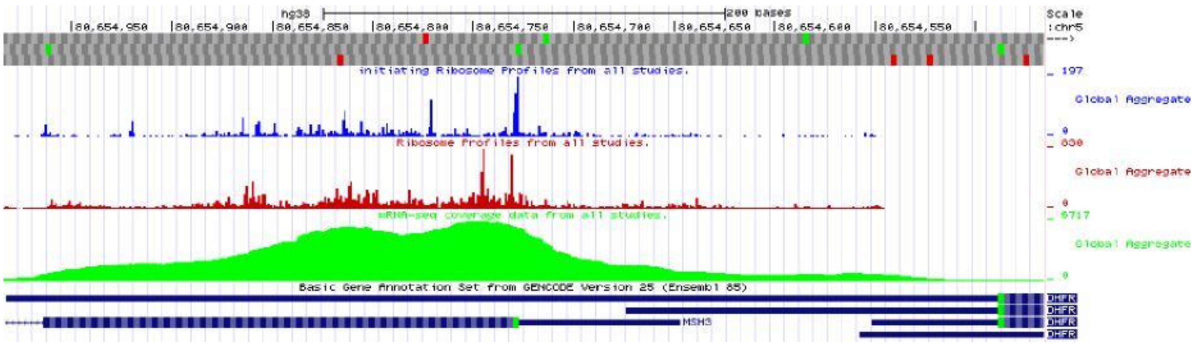

Supplementary Fig.4. 5'leader of *DHFR* gene (GENCODE 25). Plot is derived from GWIPs-Viz browser, top track is base position in 3 reading frames where red tiles are stop codons and green tiles are AUGs. Blue track is initiating ribosomes (P-sites), red track is elongating ribosomes (A-sites), green track is mRNA-seq reads. Transcript with the longest 5'leader (ENST00000439211.6) has 2 upstream AUGs overlapping with the coding exon of *MSH3* in a different strand. There is a clear signal from elongating ribosomes between *MSH3* start AUG and *DHFR* start AUG.

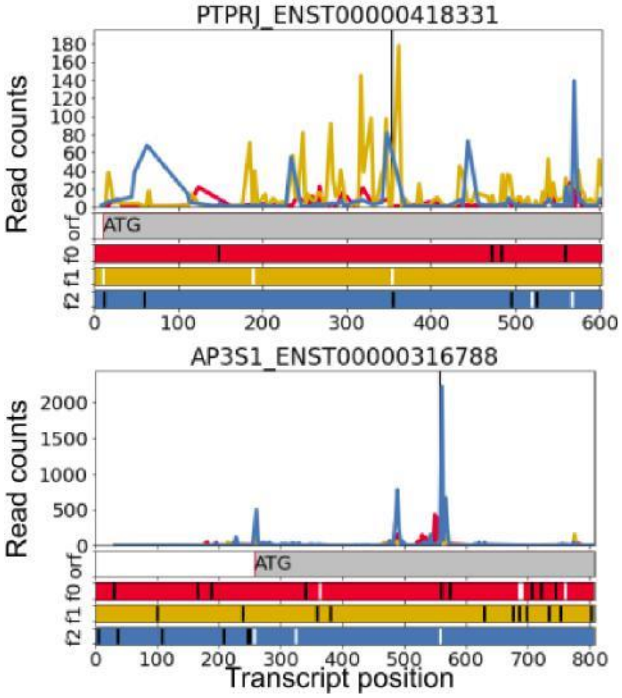

Supplementary Fig.5. Subcodon Ribo-seq profiles of 3 genes with predicted AUG-extensions. Densities of ribosome footprints differentially coloured based on the supported reading frame. The colours are matched to the reading frames in ORF plot at the bottom where AUG codons are depicted as white and stop codons as black dashes. Black vertical lines indicate the starts of the annotated CDS. Yellow rectangle highlights the predicted extension. The genomic intervals including primary extension and the first at least 150 nucleotides of CDS are shown. Source data is provided as a Source Data file.

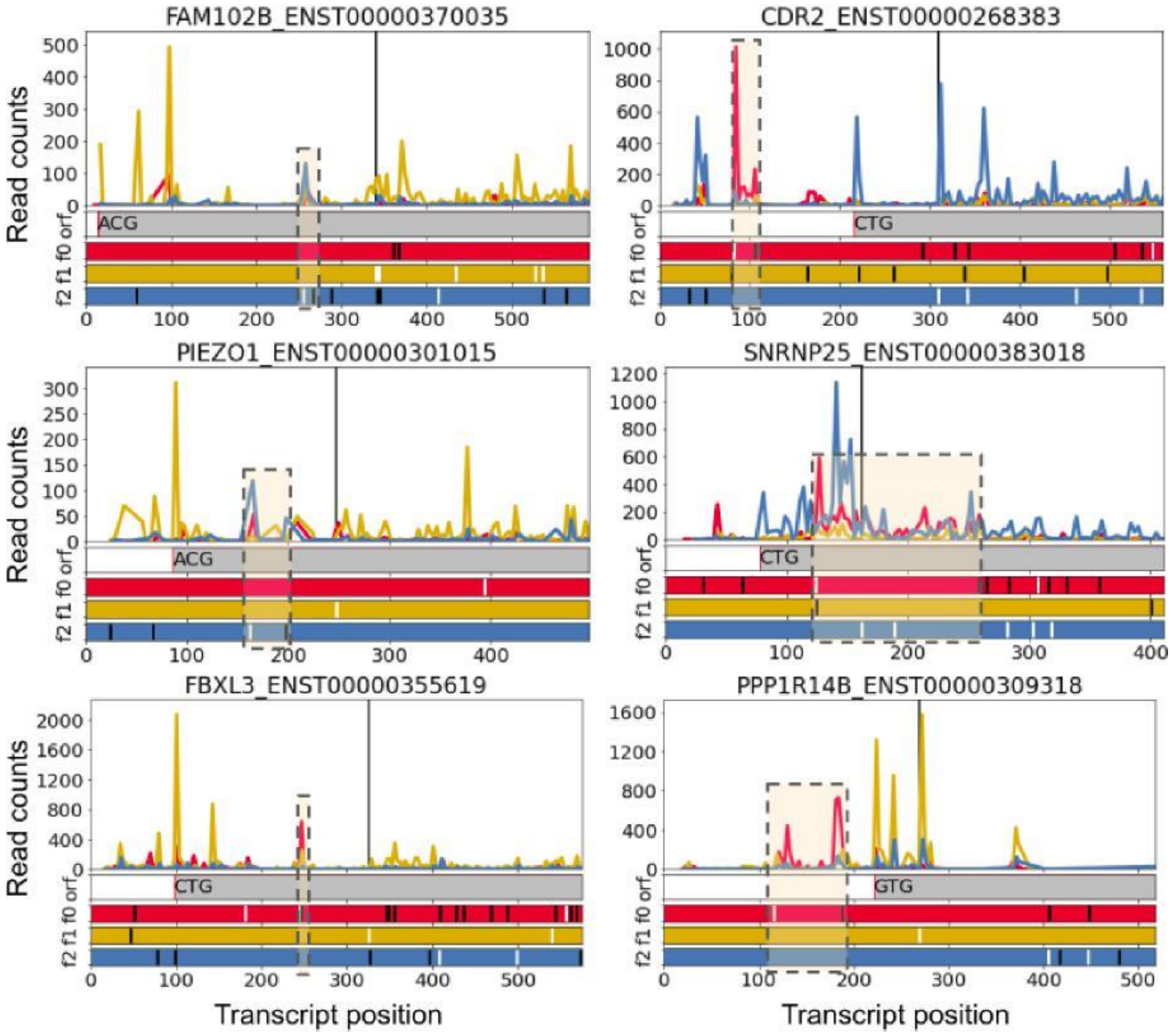

Supplementary Fig.6. Subcodon Ribo-seq profiles of 6 genes with non-AUG extended proteoforms from RiboSET which have overlapping or non-overlapping uORFs. Densities of ribosome footprints differentially coloured based on the supported reading frame. The colours are matched to the reading frames in ORF plot at the bottom where AUG codons are depicted as white and stop codons as black dashes. Black vertical lines indicate the starts of the annotated CDS. Grey bar shows predicted extension. The genomic intervals including predicted extension in the 5'leader and upstream part of CDS are shown. AUG uORFs are framed and highlighted with light orange. Source data is provided as a Source Data file.

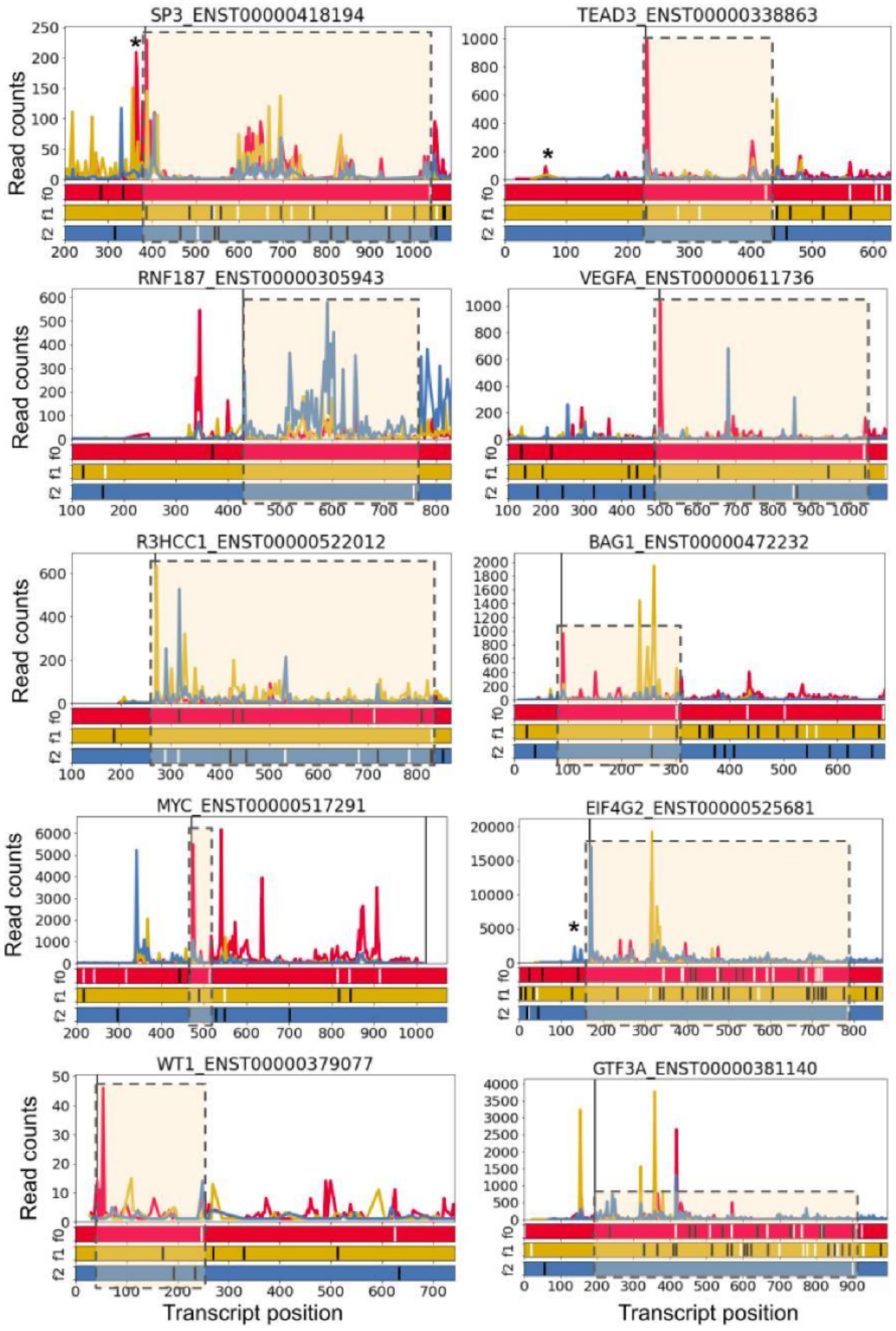

Supplementary Fig.7. Subcodon Ribo-seq profiles of genes with non-AUG extended proteoforms from [Ivanov et al 2011] which are annotated in Gencode35 (total 24, only 17 has Ribo-seq data; part1). Densities of ribosome footprints differentially coloured based on the supported reading frame. The colours are matched to the reading frames in ORF plot at the bottom where AUG codons are depicted as white and stop codons as black dashes. Black vertical lines indicate the starts of the annotated CDS. The genomic intervals including first nucleotides of CDS of non-AUG proteoform and part of 5'leader and CDS in case of AUG-proteoform are shown. Sequences from non-AUG start till the first AUG in the same are framed and highlighted with light orange. Source data is provided as a Source Data file.

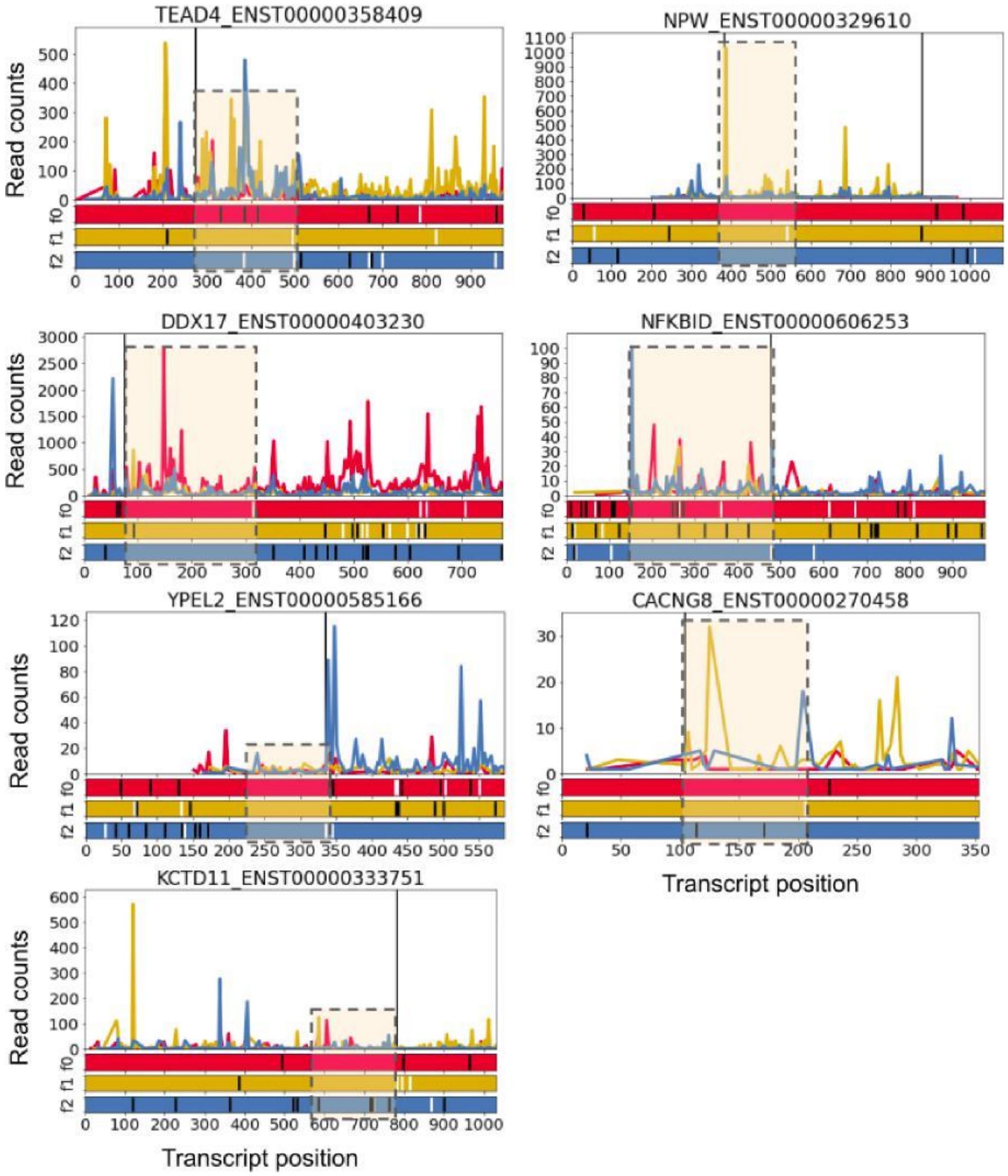

Supplementary Fig.8. Subcodon Ribo-seq profiles of genes with non-AUG extended proteoforms from [Ivanov et al 2011] which are annotated in Gencode35 (total 24, only 17 has Ribo-seq data; part2). Densities of ribosome footprints differentially coloured based on the supported reading frame. The colours are matched to the reading frames in ORF plot at the bottom where AUG codons are depicted as white and stop codons as black dashes. Black vertical lines indicate the starts of the annotated CDS. Yellow rectangle highlights the predicted extension. The genomic intervals including primary extension and the first at least 250 nucleotides of CDS are shown. The genomic intervals including first nucleotides of CDS of non-AUG proteoform and part of 5'leader and CDS in case of AUG-proteoform are shown. Sequences from non-AUG start till the first AUG in the same are framed and highlighted with light orange. Source data is provided as a Source Data file.

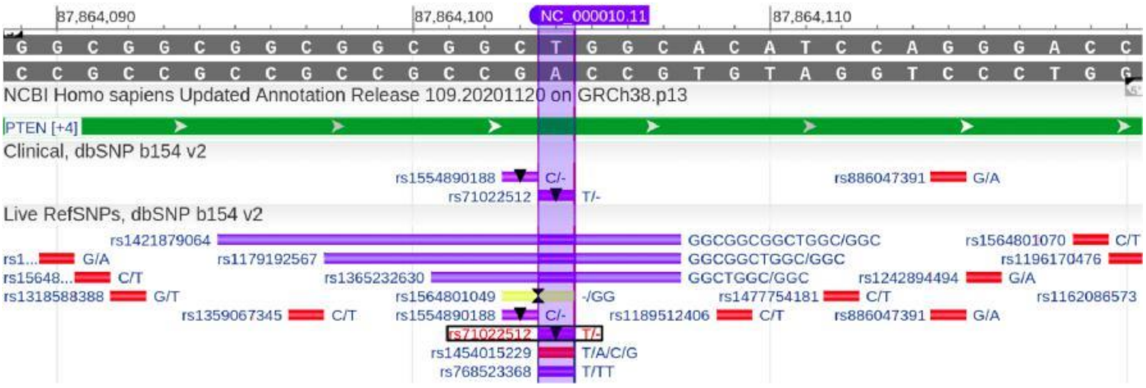

Supplementary Fig.9. Region of PTEN 5'UTR with variant rs71022512 (NC_000010.11:g.87864104del). In RefSeq CUG proteoform of PTEN is annotated and lacking T, while for both Gencode25 and Gencode35 5' leader still contains this nucleotide.

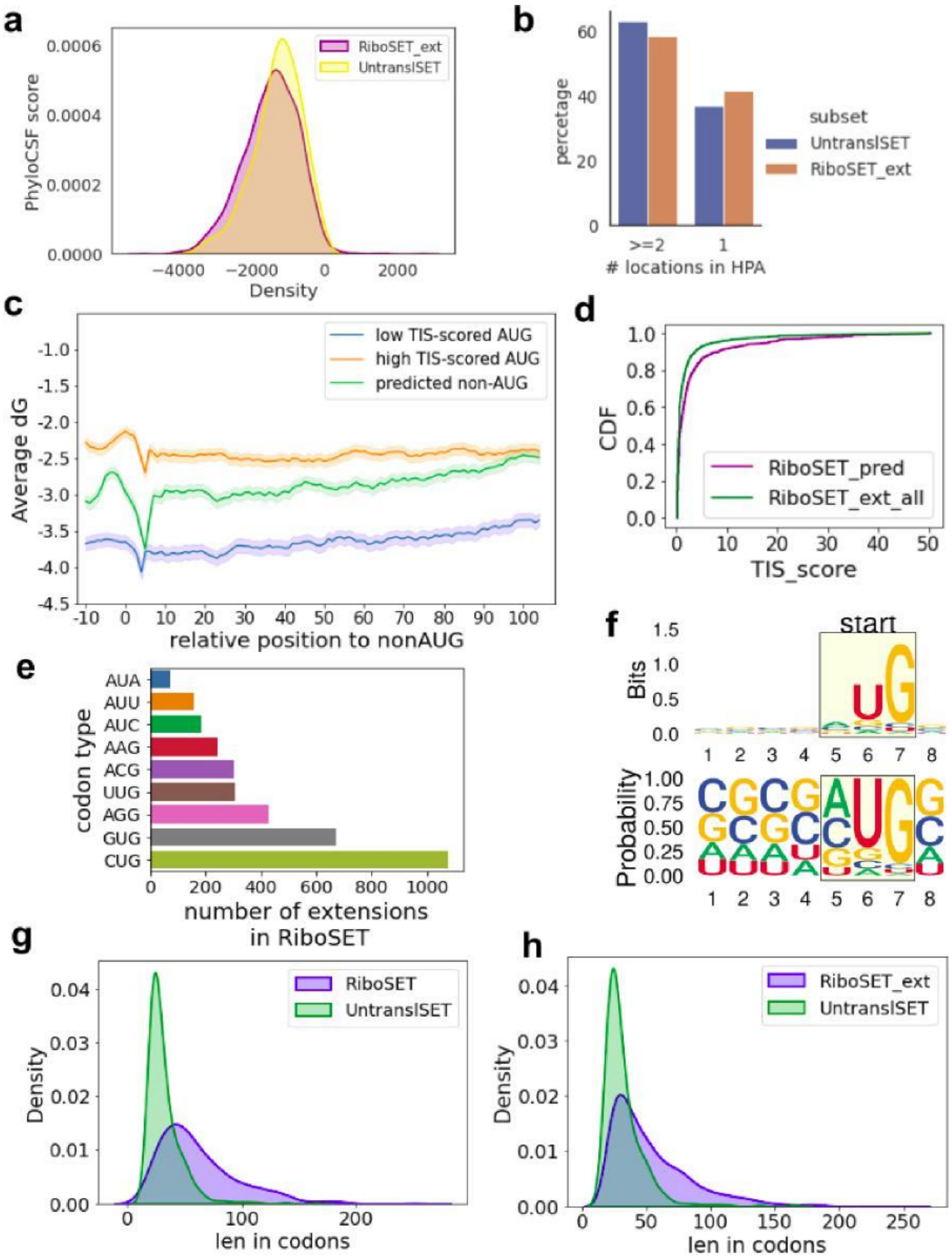

Supplementary Fig.10. Characterisation of predicted non-AUG initiation codons in RiboSET_ext. **a**. PhyloCSF score of upstream regions of RiboSET_ext and UntranslSET genes. **b.** Fraction of genes in RiboSET_ext and UntranslSET with 1 or at least 2 alternative localisations in Human Protein atlas. **c**. The stability (dG, Gibbs free energy) of mRNA secondary structure downstream of start codons within 22 nt window '0' corresponds to the start codon. 3451 starts from RiboSET_ext (green line), sample of 3500 AUG starts with high-scored TIS (blue line), sample of 3500 AUG starts with low-scored TIS (orange line). Lines are mean values across genes with 95% confidence interval. **d**. Cumulative distribution

functions of TIS scores for all non-AUG codons in theoretical NTE, RiboSET_ext (green), Trips-viz predicted non-AUG starts, RiboSET_ext (purple). **e**. Distribution of start codon types predicted by Trips-viz (RiboSET). **f**. TIS sequence logo and frequency plot of non-AUG starts predicted by Trips-viz. **g** and **h**: distribution of length (in codons) of theoretical extensions for UntranslSET, RiboSET and RiboSET_ext. Source data is provided as a Source Data file.

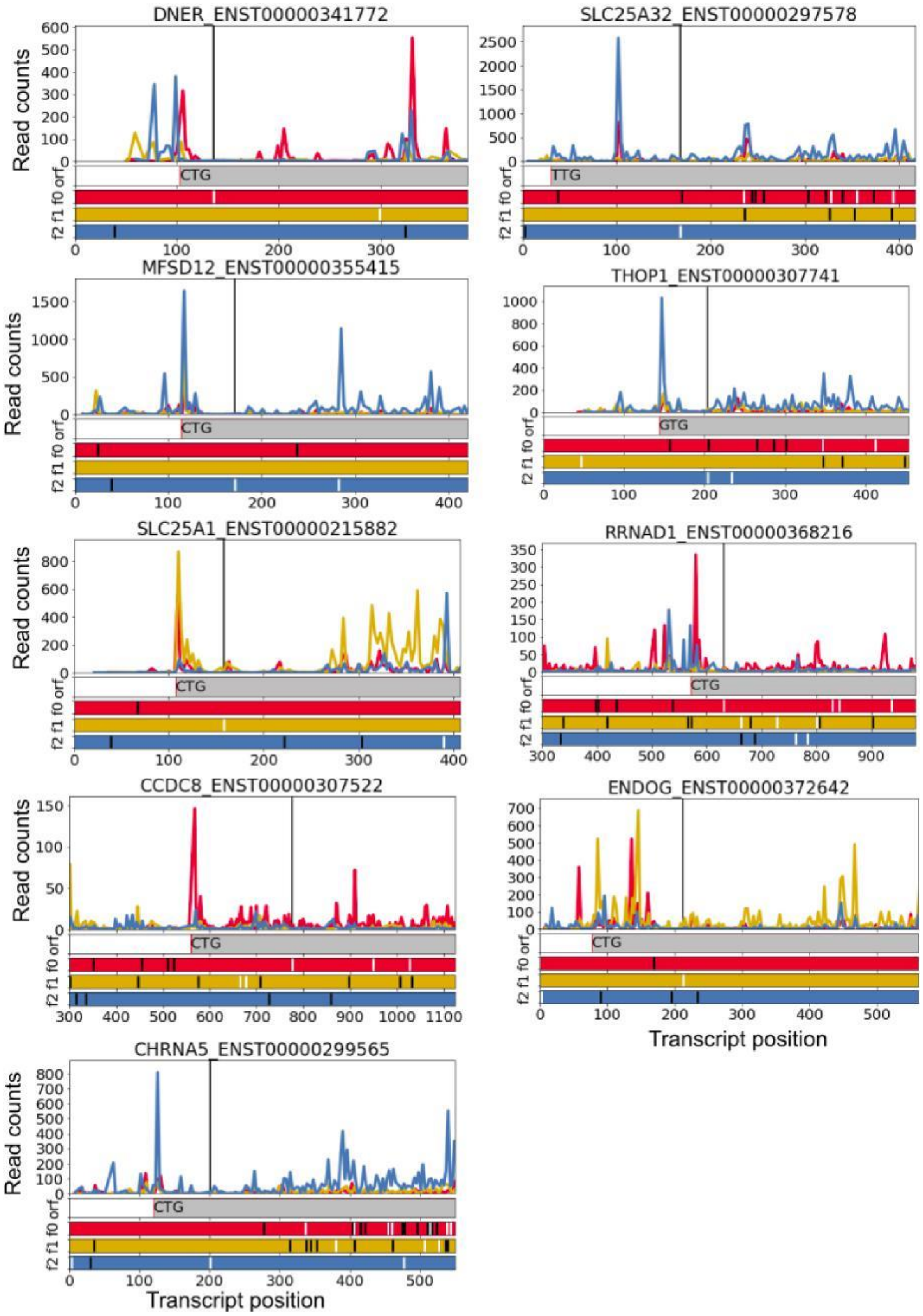

Supplementary Fig.11. Subcodon Ribo-seq profiles for examples of predicted genes with exclusive non-AUG initiation. Densities of ribosome footprints differentially coloured based on the supported reading frame. The colours are matched to the reading frames in ORF plot at the bottom where AUG codons are depicted as white and stop codons as black dashes. Black vertical lines indicate the starts of the annotated CDS. Gray bars correspond to extended CDS initiated at the proposed non-AUG starts. The genomic intervals including primary extension and the first at least 100 codons of CDS are shown. Source data is provided as a Source Data file.

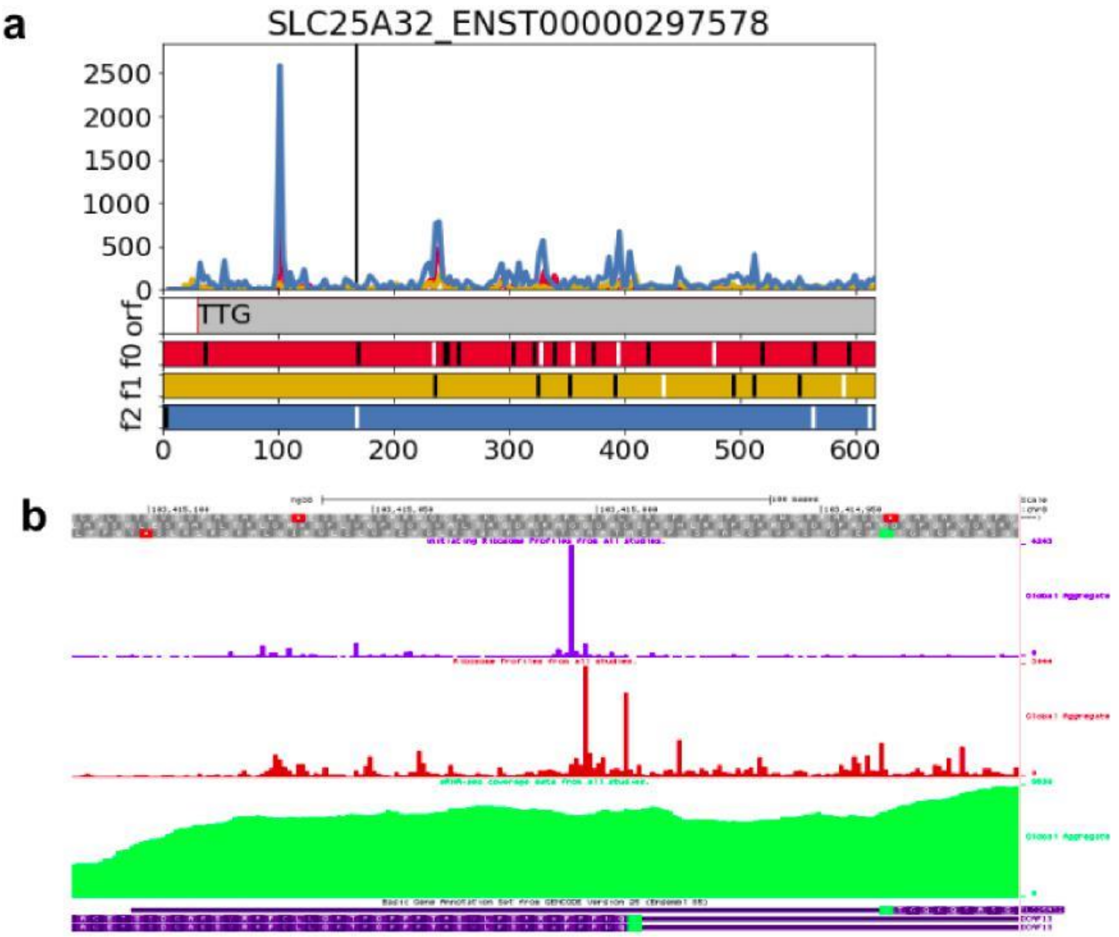

Supplementary Fig.12. Example of sole non-AUG initiation: SLC25A32. **A.** Subcodon Ribo-seq profile of SLC25A32. Densities of ribosome footprints differentially coloured based on the supported reading frame. The colours are matched to the reading frames in ORF plot at the bottom where AUG codons are depicted as white and stop codons as black dashes. Black vertical lines indicate the starts of the annotated CDS. Gray bars correspond to extended CDS initiated at the proposed non-AUG starts. The genomic intervals including primary extension and the first codons of CDS are shown. According to the Ribo-seq profile it looks like there is a second non-AUG initiation site located downstream of the predicted one. However, this high peak can be explained by overlap with CDS of DCAF13 gene. **B.** 5' leader of SLC25A32 and its overlap with DCAF13 CDS. Plot is derived from GWIPs-Viz browser, top track is base position in 3 reading frames where red tiles are stop codons and green tiles are AUGs. Blue track is initiating ribosomes (P-sites), red track is elongating ribosomes (A-sites), green track is mRNA-seq reads. Source data is provided as a Source Data file.

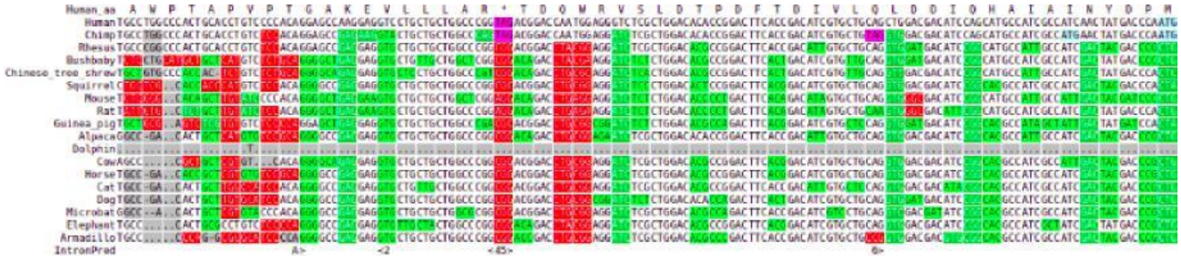

Supplementary Fig 13. Multiple genome sequence alignment for theoretical extension with upstream region for *LRP5L* gene derived with CodAlignVIew. Green colour represents synonymous or conserved substitutions, red - radical changes, purple - stop codons. cdsATG is included.

Supplementary Methods

Detection of translated regions in Trips-Viz

The Trips-Viz ranking procedure of extended proteoforms includes two steps. The first step is calculation of four parameters for each selected non-AUG extension. The first parameter Highest Reading Frame (HRF) measures the consistency of ribosome footprint triplet periodicity within the reading frame of the examined ORF by dividing the number of footprints with inferred A-sites matching the expected frame divided by the number of footprints with inferred A-sites matching the alternative best supported frame. It can be expressed as the following, [1] (**Fig.1**):

$$HRF = \{\sum f_{i,+1} \geq \sum f_{i,-1} \rightarrow \frac{\Sigma f_{i,0}}{\Sigma f_{i,+1}};\ \sum f_{i,+1} < \sum f_{i,-1} \rightarrow \frac{\Sigma f_{i,0}}{\Sigma f_{i,-1}}\},\ i \in L \quad [1]$$

Where $\boldsymbol{f_{i,0}}$, $\boldsymbol{f_{i,+1}}$, $\boldsymbol{f_{i,-1}}$ are the numbers of footprints supporting the inferred A-site codon ***i*** in a specific reading frame (0, +1, -1) within the analysed ORF of ***L*** codons in length. The positions of the A-sites were obtained directly from Trips-Viz where they are calculated using a fixed off-set from the 5' end of the footprint specific for each footprint length within the corresponding dataset [1].

The second parameter StartRiseUp (SRU, **Fig.1**) measures the increase of footprint density at the potential start, and is calculated as the following [2]: Where $\boldsymbol{f_{i,0}}$ is the number of footprints supporting codon ***i*** at the expected 0 frame. ***i***=0 is set to the start codon of the analysed ORF.

$$SRU = \ln ln \frac{1 + \sum_{i=1}^{4} f_{i,0}}{1 + \sum_{=-3}^{0} f_{i,0}} \qquad [2]$$

The third parameter is Non-Zero Coverage (NZC, **Fig.1**) which is a fraction of A-site codons where the read count at reading frame 0 is greater than the read count in the +1 or -1 frame divided of the total number of codons with at least one read in any frame. It can be expressed as the following [3]:

$$= \frac{\Sigma}{\quad} \{ \ _{,0} > \quad (\ _{,-1}, \quad _{,+1}) \rightarrow \quad = 1; \quad _{,0} \leq \quad (\ _{,-1}, \quad _{,+1}) \rightarrow \quad = 0 \}, \ \in \qquad [3]$$

Where ***L*** is the total number of codons in the ORF supported by at least one read, $\boldsymbol{N_i}$ is the binary codon index and $\boldsymbol{f_{i,0}}$, $\boldsymbol{f_{i,+1}}$, $\boldsymbol{f_{i,-1}}$ are the numbers of footprints supporting the inferred A-site codon ***i*** in a specific reading frame (0, +1, -1). NZC score measures the uniformity of footprint distribution which reduces the impact of high local peaks which are unlikely to occur as a result of productive translation.

The final parameter (AVG, [4], **Fig.1**) is the average read density. Which is calculated from the sum of the reads in the expected reading frame, divided by the length of the ORF in codons. It can be expressed as the following:

$$= \frac{\Sigma \quad _{,0}}{\quad} \ [4]$$

Where ***L*** is the length of analysed ORF in codons and $\boldsymbol{f_{i,0}}$ is the number of footprints supporting codon ***i*** at the expected 0 frame.

The second step is calculating the final ranking by summing the ranks of the individual features.

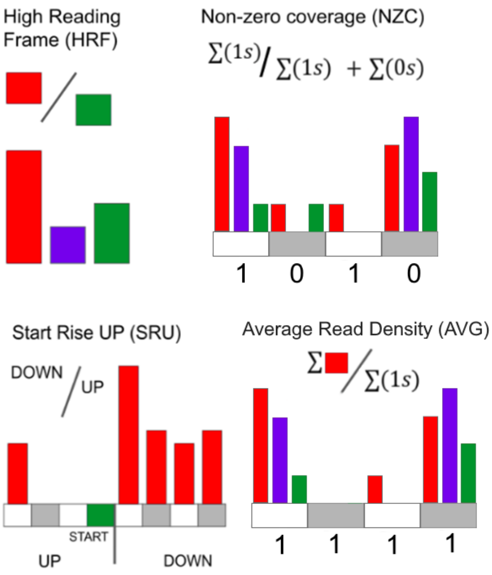

Fig.1. Features that were used for calculating ranked translated extended proteoforms in Trips-viz. Each feature was ranked individually, then all ranks were summed to produce the final rank.

In-frame and out-of-frame AUGs in theoretical extensions of PhyloSET and RiboSET

Assuming that during genome annotation it is the first AUG codon that is usually annotated as a start (in the absence of additional experimental evidence overriding this rule) we were surprised to find in-frame AUG codons in the N-terminal extensions of twelve genes from RiboSET and PhyloSET. In 8 genes (***DUSP5***, ***PDCD6***, ***CCDC127***, ***EID2***, ***DHX33***, ***SLC17A5***, ***TMX3***, ***MXD4)*** we found that their 5' leader are longer in GENCODE v25 in comparison to RefSeq ones which do not possess such upstream non-annotated AUGs. No evolutionary or translational signatures advocate for use of such in-frame AUGs in these genes. In case of ***DUSP5*** and ***PDCD6***, upstream AUG is located very close to the 5' end of the transcript (4nt and 5nt) and may not be recognised by the pre-initiation ribosomal complex. Transcripts in GENCODE v25 and v35 and RefSeq for ***TK1*** gene have an upstream AUG and a longer 5' leader with quite decent conservation (high PhyloP score) although RNA-seq data (available in GWIPs-viz [1] does not seem to support such a long 5' leader. Of note, high PhyloP score could be due to a conserved promoter that is not a part of transcript as was recently shown for ***Hoxa*** genes [2]. According to Trips-viz ribo-seq profile, the upstream AUG does not seem to have any translation signal (**Supplementary Fig.3**). Trips-viz Ribo-seq profiles of ***C1GALT1, LY6K and DHFR*** look like there might be an AUG-extension (**Supplementary Fig.3**), although AUGs in ***DHFR*** are located in the overlap with coding exons of another gene (***MSH3***) on a different strand (**Supplementary Fig.4**).

Trips-viz predicted 3 more genes with AUG extensions. ***STIM2*** (STromal Interaction Molecule 2) has clearly translated AUG-extension according to Trips-viz Ribo-seq profile while it has only UUG proteoforms in GENCODE v35 and the latest RefSeq in humans. In contrast, in mice both AUG- and UUG-proteoforms are annotated. ***PTPRJ*** (Protein Tyrosine Phosphatase, type J) has also been shown to possess AUG-extension according to

Trips-Viz predictions. An upstream AUG initiation has been described in Karagyozov et al. 2020 [3]. ***AP3S1*** (Adaptor Related Protein Complex 3 Subunit Sigma 1) is the last candidate with AUG-extension predicted by Trips-viz (**Supplementary Fig.5**).

We also addressed the occurrence of out-of-frame AUGs between non-AUGs and annotated AUGs. Out-of-frame AUG are a source of uORFs which may be involved in the regulation of relative proteoform synthesis. Translation of an out-of-frame uORF is likely to inhibit the synthesis of the extended proteoform (especially when it overlaps the extension), but may be less detrimental to the translation initiation at annotated AUG which ribosome could access via reinitiation [4,5]. On the contrary, initiation at out-of-frame AUG located within the extension is unlikely to affect the synthesis of the longer proteoform but would inhibit the synthesis of the shorter one. uORFs is a rich source of versatile mechanisms for gene-specific regulation at the translation level in response to specific conditions [6–9] and it is likely that they may also be used for regulation of a ratio between proteoforms in a similar manner. In RiboSET, 57 genes (57 transcripts) have at least one out-of-frame AUG codon in their theoretical extensions. In PhyloSET, 24 genes (36 transcripts) have at least one out-of-frame AUG codon in their theoretical extensions. We explored their Ribo-seq profiles in order to spot translated uORFs. In some genes, uORFs are located within translated extension (e.g. ***FAM102B***, ***PIEZO1, SNRNP25***, ***FBXL3***), while there are also examples where uORF is situated upstream of translated extension (e.g. ***CDR2***, ***PPP1R14B***, **Supplementary Fig.6**).

Exclusive non-AUG initiation

Most of our examples are in RiboSET exclusively and only CCDC8 is present in both RiboSET and PhyloSET. We also observed that there might be multiple non-AUG translation initiation sites with varying efficiency. For instance, the second potential initiation peak located downstream of predicted non-AUG start in ***SLC25A32*** can be explained by overlap of 5’ leader with another transcript of gene ***DCAF13***, located on a different strand

(**Supplementary Fig.12**). However, we cannot exclude the possibility of multiple non-AUG initiations, e.g for the second high peak located upstream of predicted non-AUG start in ***THOP1*** which may be attributed to GUG codon (**Supplementary Fig.11**).

Supplementary References

Michel, A. M. ***et al.*** GWIPS-viz: development of a ribo-seq genome browser. ***Nucleic Acids Res.*** **42**, D859–64 (2014).

2. Ivanov, I. P. ***et al.*** Evolutionarily conserved inhibitory uORFs sensitize mRNA translation to start codon selection stringency. ***Proc. Natl. Acad. Sci. U. S. A.*** **119**, (2022).

3. Karagyozov, L., Grozdanov, P. N. & Böhmer, F.-D. The translation attenuating arginine-rich sequence in the extended signal peptide of the protein-tyrosine phosphatase PTPRJ/DEP1 is conserved in mammals. ***PLoS One*** **15**, e0240498 (2020).

4. Gunišová, S., Hronová, V., Mohammad, M. P., Hinnebusch, A. G. & Valášek, L. S. Please do not recycle! Translation reinitiation in microbes and higher eukaryotes. ***FEMS Microbiol. Rev.*** **42**, 165–192 (2018).

5. Skabkin, M. A., Skabkina, O. V., Hellen, C. U. T. & Pestova, T. V. Reinitiation and other unconventional posttermination events during eukaryotic translation. ***Mol. Cell*** **51**, 249–264 (2013).

6. Andreev, D. E. ***et al.*** Oxygen and glucose deprivation induces widespread alterations in mRNA translation within 20 minutes. ***Genome Biol.*** **16**, 90 (2015).

7. Andreev, D. E. ***et al.*** Translation of 5’ leaders is pervasive in genes resistant to eIF2 repression. ***Elife*** **4**, e03971 (2015).

8. Starck, S. R. ***et al.*** Translation from the 5’ untranslated region shapes the integrated stress response. ***Science*** **351**, aad3867 (2016).

9. Mueller, P. P. & Hinnebusch, A. G. Multiple upstream AUG codons mediate translational control of GCN4. ***Cell*** **45**, 201–207 (1986).
