# Supplementary material for: Thousands of human non-AUG extended proteoforms lack evidence of evolutionary selection among mammals
Source: Nat Commun. 2022 Dec 23;13:7910. doi: 10.1038/s41467-022-35595-6 (PMC9789052; doi:10.1038/s41467-022-35595-6)
Supplement: Supplementary file 3 — Description of Additional Supplementary Files [file 41467_2022_35595_MOESM3_ESM.pdf]

**Description of Additional Supplementary Files**

File Name: Supplementary Data 1
Description: A_PhyloSET B_RiboSET C_UntranslSET

File Name: Supplementary Data 2
Description: RiboSET_ext

File Name: Supplementary Data 3
Description: overlap with Ivanov et al, Yeom et al, Van Damme et al

File Name: Supplementary Data 4
Description: TIS score for starts in RiboSET

File Name: Supplementary Data 5
Description: Gene Ontology for RiboSET and PhyloSET

File Name: Supplementary Data 6
Description: Human Protein Atlas, TargetP, SignalP and DeepTMHMM prediction of localisation for PhyloSET, RiboSET, RiboSET_ext and UntranslSET

File Name: Supplementary Data 7
Description: gene with exclusive non-AUG initiation

File Name: Supplementary Data 8
Description: Ribo-seq studies with triplet periodicity scores

File Name: Supplementary Data 9
Description: ClinVar variants within RiboSET_ext extensions
